# Preliminary review on the prevalence, proportion, geographical distribution, and characteristics of naturally acquired *Plasmodium cynomolgi* infection in mosquitoes, macaques, and humans: a systematic review and meta-analysis

**DOI:** 10.1186/s12879-021-05941-y

**Published:** 2021-03-12

**Authors:** Manas Kotepui, Frederick Ramirez Masangkay, Kwuntida Uthaisar Kotepui, Giovanni De Jesus Milanez

**Affiliations:** 1 Medical Technology, School of Allied Health Sciences, Walailak University, Tha Sala, Nakhon Si Thammarat, Thailand; 2 Department of Medical Technology, Institute of Arts and Sciences, Far Eastern University-Manila, Manila, Philippines

**Keywords:** *Plasmodium*, Malaria, Zoonoses, Mosquitoes, Monkey, Macaques

## Abstract

**Background:**

*Plasmodium cynomolgi* is a simian malaria parasite that has been reported as a naturally acquired human infection. The present study aims to systematically review reports on naturally acquired *P. cynomolgi* in humans, mosquitoes, and macaques to provide relevant data for pre-emptive surveillance and preparation in the event of an outbreak of zoonotic malaria in Southeast Asia.

**Methods:**

The protocol of the systematic review was registered at PROSPERO with approval ID CRD42020203046. Three databases (Web of Science, Scopus, and MEDLINE) were searched for studies reporting the prevalence of *P. cynomolgi* infections in Southeast Asian countries between 1946 and 2020. The pooled prevalence or pooled proportion of *P. cynomolgi* parasitemia in humans, mosquitoes, and macaques was estimated using a random-effects model. Differences in the clinical characteristics of *P. cynomolgi* infections were also estimated using a random-effects model and presented as pooled odds ratios (ORs) or mean differences (MDs) with 95% confidence intervals (CIs).

**Results:**

Thirteen studies reporting on the prevalence of naturally acquired *P. cynomolgi* in humans (3 studies, 21 cases), mosquitoes (3 studies, 28 cases), and macaques (7 studies, 334 cases) were included. The results demonstrated that the pooled proportion of naturally acquired *P. cynomolgi* in humans was 1% (95% CI, 0.1%, I^2^, 0%), while the pooled proportion of *P. cynomolgi* infecting mosquitoes was 18% (95% CI, 10–26%, I^2^, 32.7%). The pooled prevalence of naturally acquired *P. cynomolgi* in macaques was 47% (95% CI, 27–67%, I^2^, 98.3%). Most of the cases of naturally acquired *P. cynomolgi* in humans were reported in Cambodia (62%) and Malaysia (38%), while cases of *P. cynomolgi* in macaques were reported in Malaysia (35.4%), Singapore (23.2%), Indonesia (17.3%), Philippines (8.5%), Laos (7.93%), and Cambodia (7.65%). Cases of *P. cynomolgi* in mosquitoes were reported in Vietnam (76.9%) and Malaysia (23.1%).

**Conclusions:**

This study demonstrated the occurrence of naturally acquired *P. cynomolgi* infection in humans, mosquitoes, and macaques. Further studies of *P. cynomolgi* in asymptomatic human cases in areas where vectors and natural hosts are endemic are extensively needed if human infections with *P. cynomolgi* do become public health problems.

**Supplementary Information:**

The online version contains supplementary material available at 10.1186/s12879-021-05941-y.

## Background

*Plasmodium* spp. infections in humans have been identified with *Plasmodium falciparum*, *P. malariae*, *P. vivax*, *P. ovale*, and *P. knowlesi* [1]. Although *P. falciparum* and *P. vivax* are the two major malaria species that cause malaria in humans worldwide, the infection of *P. knowlesi* is a major cause of simian malaria in Malaysian Borneo [1–4] and is also reported as a cause of simian malaria in other parts of Southeast Asia, including Indonesia [5], Laos [6], Vietnam [7, 8], and Thailand [9]. *P. knowlesi* and other simian malaria parasites, including *P. fieldi*, *P. coatneyi*, *P. cynomolgi*, and *P. inui,* mainly infect long-tailed (*Macaca fascicularis*) and pig-tailed macaques (*Macaca nemestrina*) [2].

*P. cynomolgi* has accidentally and experimentally been reported as a cause of human malaria [3–5], but there was a case report showing this parasite as a naturally acquired human infection in a Malay woman from the east coast of Peninsular Malaysia who lives in an area where long-tailed macaques are present [6]. *P. cynomolgi* was first observed in 1907 in *Macaca fascicularis* collected in Java [7]. *P. cynomolgi* has morphological features similar to *P. vivax,* as shown by microscopy, including the asexual cycle (48 h), prepatent periods, and presence of hypnozoites, which can initiate relapses [6]. The identification of *P. cynomolgi* relies on the amplification of the 18S ribosomal RNA (*rRNA*) gene by nested polymerase chain reaction (PCR) sequencing. As *P. cynomolgi* is recorded as the most recently discovered simian malaria parasite infecting humans, its prevalence, proportion, geographical distribution, and characteristics remain unclear. The present study aimed to systematically review reports on naturally acquired *P. cynomolgi* in humans, mosquitoes, and macaques to provide relevant data for pre-emptive surveillance and preparation in the event of an outbreak of zoonotic malaria in Southeast Asia.

## Methods

### Protocol for reporting the systematic review and meta-analysis

The systematic review and meta-analysis followed the Preferred Reporting Items for Systematic Reviews and Meta-Analyses (PRISMA) guidelines [8]. The protocol of the systematic review was registered at the PROSPERO International Prospective Register of Systematic Reviews with approval ID CRD42020203046.

### Search strategy

Studies reporting the prevalence of *P. cynomolgi* parasitemia in humans, mosquitoes, and macaques in Southeast Asian countries between 1946 and 2020 were systematically searched in three databases: Web of Science, Scopus, and MEDLINE. The search term used to find the potentially relevant studies was “cynomolgi”. The searches of the reference list in the included studies or the review articles and the searches of the additional source(s) such as Google Scholar were performed to maximize the number of the included studies and to prevent any missing studies during the searches of the main databases.

### The eligibility criteria and study selection

The inclusion criterion for selection in the present study was that any included studies must be primary studies reporting the prevalence or incidence of *P. cynomolgi* parasitemia in humans, mosquitoes, and macaques. The exclusion criteria were (1) studies without data of interest; (2) studies including populations outside Southeast Asian countries where sufficient reports on *P. cynomolgi* could be retrieved; (3) studies without full text; and (4) studies published as case reports or case series, reviews, editorials, letters to the editor, in vitro/experimental studies, mosquito experiments, animal experiments, human experiments, or studies identifying *P. cynomolgi* in the same participants. Two authors (MK, FRM) independently screened the studies according to the eligibility criteria for potentially relevant studies. Any disagreement between the two authors was resolved through discussion or consultation with the third author (KUK) for the finalization of the study inclusion.

### Data extraction

Data from the included studies were extracted for pilot standardization. The following data of each study were extracted: author name, year of publication, year of study, study site, types of participant or sample (humans, monkeys, or mosquitoes), age, gender ratio, types of PCR used for *P. cynomolgi* identification, target gene for PCR, number of *Plasmodium* species identified, and parasite density. Data extraction was performed by one author (MK) and cross-checked by two authors (FRM and GDM).

### Quality of the included studies

The methodological quality of the included studies was assessed using the adapted version of the Newcastle-Ottawa Scale (NOS) [9] with a maximum of three scores (Is the Case Definition Adequate?, Representativeness of the Cases, and Ascertainment of Exposure). For the present study, studies achieving a NOS score of two or greater were considered high-quality studies.

### Data synthesis

The pooled prevalence or the pooled proportion of *P. cynomolgi* parasitemia compared to all *Plasmodium* species or all cases enrolling humans, mosquitoes, and macaques was estimated using a random-effects model. In the case of a very low prevalence of *P. cynomolgi* infection compared to the number of participants enrolled, the pooled proportion instead of the pooled prevalence was used to estimate the proportion of *P. cynomolgi* parasitemia per *Plasmodium* species identified in the same participants. The difference in the geographical distribution was visualized by mapping the location(s) of the *P. cynomolgi* parasitemia provided by the included studies. Differences in the clinical characteristics of *P. cynomolgi* infections between the included studies were estimated using a random-effects model and demonstrated as pooled odds ratios (ORs) or mean differences (MDs) with 95% confidence intervals (CIs). The heterogeneity across the included studies was assessed using Cochrane’s Q and I^2^ (inconsistency) statistics.

### Publication bias

Publication bias across the included studies was assessed using the funnel plot and Egger’s test. In the absence of publication bias, the funnel plot should approximately resemble a symmetrical funnel, while an asymmetrical appearance indicates a small-study effect that will overestimate or underestimate the pooled effect [10].

## Results

### Search results

**Fig. 1:**
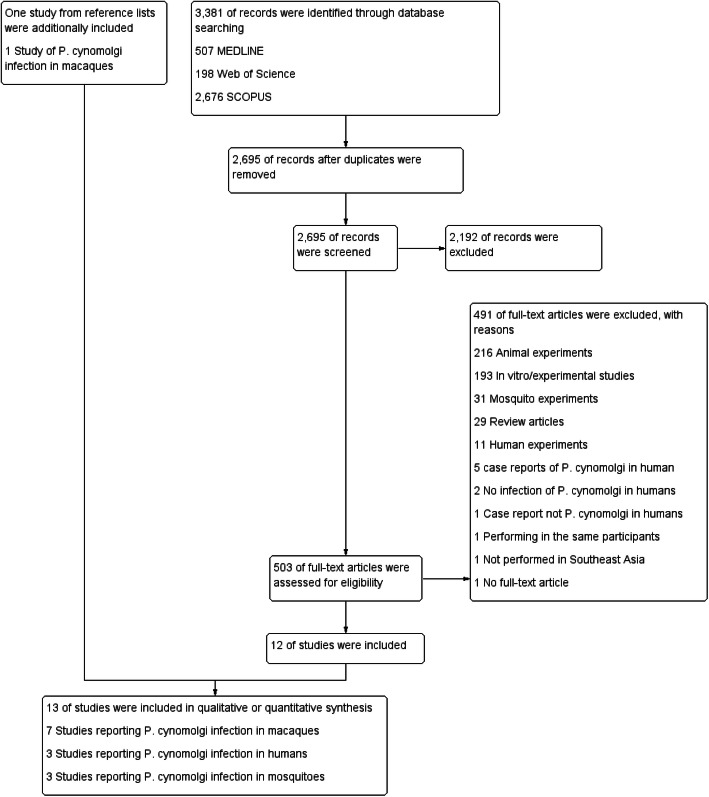
The study flow diagram. Twelve studies were from the database searches, and one study was from the searches of review articles

The initial search yielded 3381 articles (Fig. 1). Out of those articles, 686 articles were removed as they were duplicates. After screening the titles and abstracts of the remaining 2695 articles, 2192 articles were excluded as they did not meet the inclusion criterion. Following a full-text review of 503 articles, a further 491 articles were excluded with reason: 216 were animal experiments, 193 were in vitro/experimental studies, 31 were mosquito experiments, 29 were review articles, 11 were human experiments, 5 were case reports of *P. cynomolgi* in humans, 2 had no infection of *P. cynomolgi* in humans, 1 was a case report not on *P. cynomolgi* in humans, 1 involved performing identification in the same participants, 1 was not performed in Southeast Asia, and 1 was not a full-text article. Therefore, 12 articles were included in the study. Another relevant article was retrieved from the searches of the reference lists of included articles or review articles. Finally, 13 studies were included in the present study. Out of the 13 studies selected, 7 studies [11–17] reporting on *P. cynomolgi* infection in macaques, 3 studies [18–20] reporting on *P. cynomolgi* infection in humans, and 3 studies [21–23] reporting on *P. cynomolgi* infection in mosquitoes were included in the final analysis.

### Characteristics of the included studies

The characteristics of the included studies are shown in Table 1. Thirteen studies [11–23] reporting the prevalence or incidence of naturally acquired *P. cynomolgi* in humans (3 studies, 21 cases), mosquitoes (3 studies, 26 cases), and macaques (7 studies, 334 cases) were included for qualitative and quantitative syntheses. Details of *P. cynomolgi* mono and mixed infections in humans, *Anopheles*, and macaques are shown in Table 2. Among the 3 studies reporting on *P. cynomolgi* in humans between 2013 and 2017, one study was conducted in Northern Sabah of Malaysia [18], one in Pailin and Battambang provinces of Cambodia [19], and one in Sarawak of Malaysia [20]. Two studies [18, 19] enrolled participants in the communities, while another study [20] enrolled malaria-positive cases for their studies. All studies used semi-nested or nested PCR with sequencing to identify *Plasmodium* species infection. All three studies [18–20] used semi-nested or nested PCR for amplification of the *SSU rRNA* gene. PCR amplification of the *cytochrome C oxidase* gene was used for DNA sequencing to confirm *P. cynomolgi* and *P. knowlesi* coinfections in one study [20]. Of the 21 cases of people who were infected with *P. cynomolgi*, 16 cases (76.2%) were male. The age of patients was 43 and 63 years in a study by Grignard et al. [18], median 28 years (range 7–64 years) in a study by Imwong et al. [19], and median 43 years (range 17–65 years) in a study by Raja et al. [20]. *P. cynomolgi* mono-infection was in 13 cases (61.9%), while *P. cynomolgi* mixed infection with other *Plasmodium* species was in 8 cases (38.1%). Mixed infections included *P. cynomolgi* with *P. knowlesi* (6 cases, 75%) and with *P. vivax* (2 cases, 25%). Only one study [18] reported on the occupation of patients and also observed monkeys. Naturally acquired *P. cynomolgi* in humans was reported in Cambodia (62%) and Malaysia (38%). Details of *P. cynomolgi* infections in humans are shown in Table 3.

**Table 1:** Characteristics of the included studies

No.	Authors	Year of study	Study site	Types of samples/number of samples	Number of malaria cases by PCR	Number of ***P. cynomolgi*** cases by PCR	Age of patients with ***P. cynomolgi*** (years)	Number of male patients with ***P. cynomolgi***	PCR for ***P. cynomolgi***	Target gene for PCR
1.	Grignard et al., 2019 [18]	2015	Northern Sabah, Malaysia	Human/876	54	2	43, 63	2 (100%)	Genus specific semi-nested PCR	*18S rRNA*
2.	Imwong et al., 2019 [19]	2013–2016	Pailin and Battambang provinces, Cambodia	Human/14,732	1361	13	Median 28, range 7–64	11 (84.6%)	Nested PCR	*18S rRNA*
3.	Raja et al., 2020 [20]	2013–2017	Kapit, Sarawak, Malaysia	Human/1047 (seropositive for malaria)	845	6	36, 18, 63, 50, 17, 65	3 (50%)	Nested PCR	*18S rRNA*
4.	Chinh et al., 2019 [21]	2005–2018	Southern Vietnam	Mosquitoes/1386	40	9			Nested PCR	*18S rRNA*
5.	Chua et al., 2017 [22]	2013–2014	Kudat district, Sabah, Malaysia	Mosquitoes/1586	23	6			Nested PCR	*18S rRNA*
6.	Maeno et al., 2015 [23]	2010–2013	Vietnam	Mosquitoes/6062	86	11			Nested PCR	*18S rRNA*
7.	Akter et al., 2015 [11]	2014	Selangor, Malaysia	Macaques/70	36	18			Nested PCR	*18S rRNA*
8.	Amir et al., 2020 [12]	2016	Johor, Malaysia	Macaques/103	64	42			Nested PCR	*18S rRNA*
9.	Lee et al., 2011 [15]	2004–2008	Sarawak, Malaysia	Macaques/108	101	61			Nested PCR	*18S rRNA*
10.	Muehlenbein et al., 2015 [16]	Not specified	Sabah, Malaysia	Macaques/41	41	4			Not specified type of PCR	*cytochrome b*
11.	Gamalo et al., 2019 [13]	2017	Luzon, Philippines	Macaques/95	95	26			Nested PCR	*18S rRNA*
12.	Zhang et al., 2016 [17]	Not specified	Batangas/Zamboanga, Philippines	Macaques/68	7	4			Nested PCR	*18S rRNA*
			Southern Sumatra, Indonesia	Macaques/50	49	48				
			Bintan Island, Indonesia	Macaques/20	16	13				
			Singapore	Macaques/40	31	26				
			Vanny, Cambodia	Macaques/54	44	27				
			Guidong, Laos	Macaques/44	30	28				
13.	Li Meizhi I, 2011 [14]	Not specified	Singapore	Macaques/92	66	56			Nested PCR	*18S rRNA*

**Table 2:** *P. cynomolgi* mono and mixed infections in humans, *Anopheles*, and macaques

	Infection		Case total	Location total
	Mono	Mixed		
**Human (zoonotic)**			21	
**Malaysia, Northern Sabah**				Malaysia
a. Kudat	2	0		8
b. Kota Marudu				
c. Pitas				
d. Ranau				
**Malaysia, Sarawak**				
e. Kapit	0	6		
**Cambodia**				Cambodia
f. Pailin and Battambang	11	2		13
**Anopheles (vector)**			28	
**Vietnam** **Southern Vietnam**				Vietnam
a. Gia Lai	6	3		20
b. Phu Yen				
c. Khanh Hoa				
d. Ninh Thuan				
e. Binh Thuan				
f. Dong Nai				
g. Binh Phuoc				
h. Khanh Phu, Khanh Vinh district i. Khanh Hoa	6	5		
**Malaysia, Sabah**				Malaysia
j. Kudat	4	4		8
**Macaques (natural host)**			334	
**Malaysia**				Malaysia
a. Hulu Selangor	2	16		125
*B. Pahang*	15	27		
c. Perak				
d. Johor				
e. Kapit	1	60		
f. Sabah	4	0		
**Philippines**				Philippines
g. Palawan	0	23		27
h. Batangas	2	1		
i. Zamboanga	1	0		
**Indonesia**				Indonesia
j. South Sumatra	39	9		61
k. Bintan Island	9	4		
**Singapore**				Singapore
l. Singapore	23	3	(40 cases did not report species of infection)	66
**Cambodia**				Cambodia
m. Vanny	24	3		27
**Laos**				Laos
n. Guidong	28	0		28

**Table 3:** Details of *P. cynomolgi* infections in humans

***N*** = 21 (mono/mixed)	Combination	Prevalence per all malaria cases (2260 cases)	Regional trend
Mono	–	13 (0.58)	Northern Sabah, Pailin, and Battambang
Dual	*P. cynomolgi* + *P. knowlesi*	6 (0.27)	Kapit, Sarawak
	*P. cynomolgi* + *P. vivax*	2 (0.09)	Pailin and Battambang

Among the 3 studies reporting the *P. cynomolgi* infection in mosquitoes between 2010 and 2018, 2 studies [21, 23] were conducted in South or South-Central Vietnam, while another study [22] was conducted in Malaysia. All studies used nested PCR with sequencing to identify *Plasmodium* species infection. One study identified *Plasmodium* infection in four *Anopheles,* including *An. dirus*, *An. maculatus*, *An. aconitus*, and *An. minimus* [21]. One study identified *Plasmodium* infection in *An. dirus* [23]. Another study identified *Plasmodium* infection in *An. balabacensis Baisas* [22]. *P. cynomolgi* mono-infection occurred in 16 cases (57.1%), while *P. cynomolgi* mixed infection with other *Plasmodium* species occurred in 12 cases (42.9%). Mixed infections with *P. cynomolgi* were dual (7 cases, 58.3%), triple (4 cases, 33.3%), and quadruple (1 case, 8.3%) infections. Most of the *P. cynomolgi* infected cases were mixed infections with *P. inui* (5 cases, 41.7%), *P. vivax* (5 cases, 41.7%), *P. knowlesi* (2 cases, 16.7%), *P. coatneyi* (1 case, 8.33%), and *P. fieldi* (1 case, 8.33%).

Among the 7 studies reporting on 334 macaques infected with *P. cynomolgi* between 2004 and 2019, 4 studies (57.1%) [11, 12, 15, 16] were conducted in Malaysia, 1 in the Philippines [13], and 1 in Singapore [14]. Another study [17] was conducted in five countries, including the Philippines, Indonesia, Singapore, Cambodia, and Laos. Four studies [11, 13, 14, 17] identified *Plasmodium* species in long-tailed macaques, while 3 studies [12, 14, 16] identified *Plasmodium* species in both long-tailed and pig-tailed macaques. All studies used nested PCR for amplification of the *18S rRNA* gene except a study by Muehlenbein et al. [16], which used nested PCR for amplification of the *cytochrome b* (*cytb*) gene. One study did not report the status of *P. cynomolgi* mono or mixed infection [16]. *P. cynomolgi* mono-infection occurred in 148 cases (50.3%), while *P. cynomolgi* mixed infection with other *Plasmodium* species occurred in 146 cases (49.7%). Mixed infections with *P. cynomolgi* were dual (14 cases, 9.6%), triple (37 cases, 25.3%), quadruple (56 cases, 38.4%), and quintuple (9 cases, 6.16%) infections. Most of the *P. cynomolgi* cases were mixed infections with *P. inui* (132 cases, 90.4%), *P. coatneyi* (75 cases, 51.4%), *P. knowlesi* (67 cases, 45.9%), and *P. fieldi* (31 cases, 21.2%). Naturally acquired *P. cynomolgi* in macaques was reported in Malaysia (125/338, 37%), Philippines (30/338, 8.88%), Indonesia (61/338, 18.1%), Singapore (82/338, 24.3%), Cambodia (27/338, 7.99%), and Laos (28/338, 8.28%).

### Quality of the included studies and publication bias

The methodological quality of the included studies was assessed using the adapted version of the NOS (Table S1). Overall, all 13 studies achieving NOS scores of three were reviewed. Publication bias among the included studies could not be assessed using the funnel plot and Egger’s test due to the low number of the included studies, as the analysis required a minimum of 10 studies for each group of participants enrolled [10].

### Geographical distribution of *P. cynomolgi* infection in humans, mosquitoes, and macaques

**Fig. 2:**
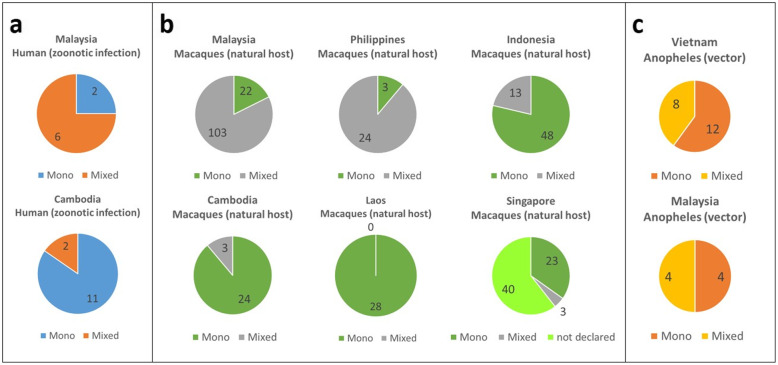
The proportion of *P. cynomolgi* cases in; **a** human, **b** *Anopheles*, and **c** macaques per Southeast Asian country. *P. cynomolgi* infection in humans has been demonstrated in Malaysia and Cambodia. *P. cynomolgi* infection in *Anopheles* was demonstrated in Vietnam and Malaysia. *P. cynomolgi* infection in macaques was demonstrated in Malaysia, the Philippines, Indonesia, Cambodia, Laos, and Singapore. Note the higher proportion (*n* = 40) of undeclared speciation in Singapore compared to mono or mixed cases combined

**Fig. 3:**
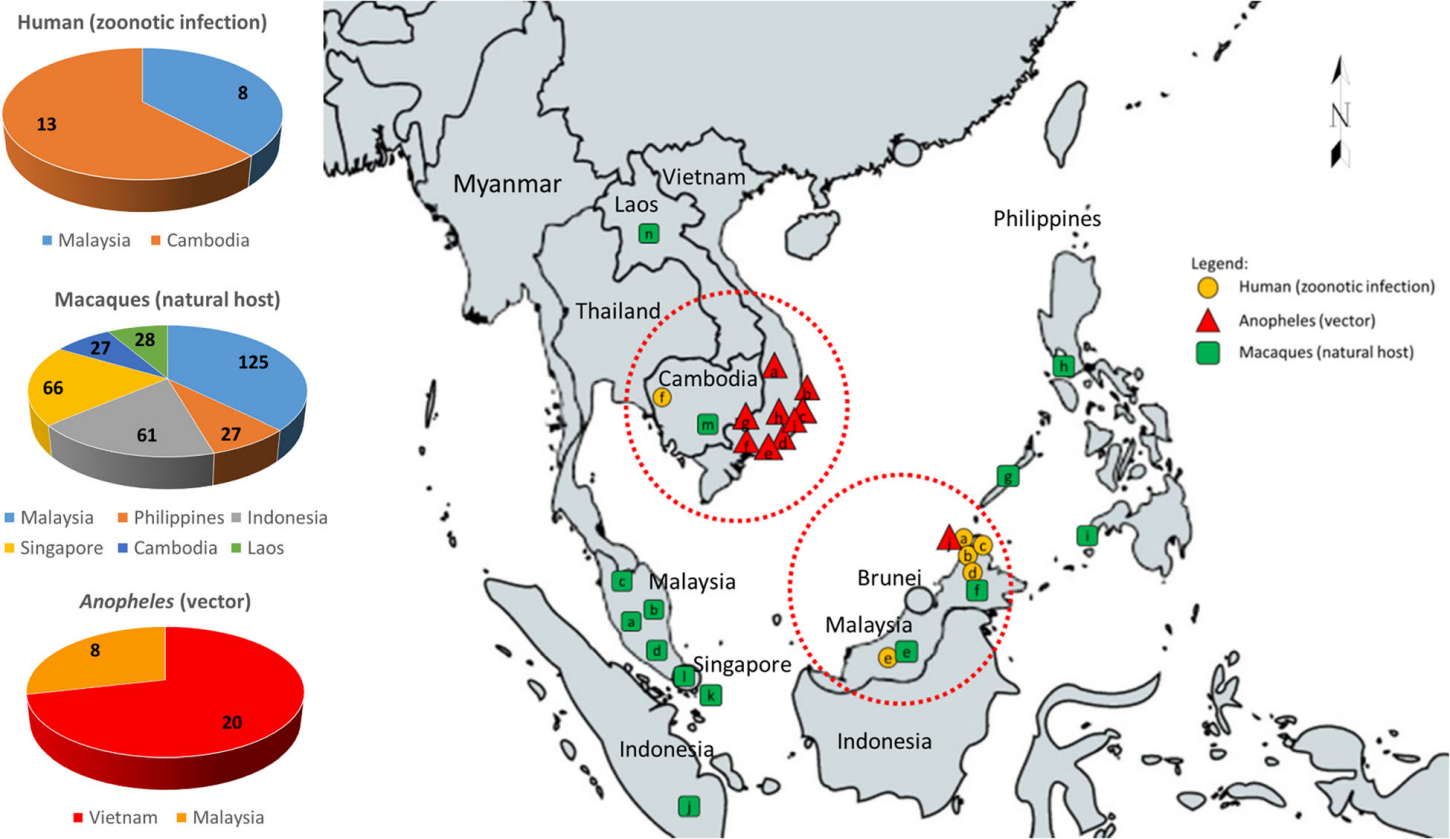
Geographic mapping and distribution of *P. cynomolgi* cases in humans, Anopheles, and macaques in Southeast Asia. Regions with human cases (red circles) were observed to have the presence of both vector and natural host. Human (zoonotic infection) **a** Kudat, **b** Kota Marudu, **c** Pitas, and **d** Ranau districts, Northern Sabah, and **e** Kapit, Sarawak, Malaysia, **f** Pailin and Battambang provinces, Cambodia; Macaques (natural host) **a** Hulu Selangor district, Selangor **b** Pahang, **c** Perak, **d** Johor, **e** Kapit, Sarawak, and **f** Sabah, Malaysia, **g** Palawan, **h** Batangas, and **i** Zamboanga, Philippines, **j** Southern Sumatra, and **k** Bintan Island, Indonesia, **l** Singapore, **m** Vanny, Cambodia; Anopheles (vector) **a** Gia Lai Province, **b** Phu Yen Province, **c** Khanh Hoa Province, **d** Ninh Thuan Province, **e** Binh Thuan Province, **f** Dong Nai Province, **g** Binh Phuoc Province, Southern Vietnam, **h** Khanh Phu, Khanh Vinh district, **i** Khanh Hoa province, South-central Vietnam, **j** Kudat district, Sabah, Malaysia. The map was generated by authors using the map freely available at https://mapchart.net/. Authors are allowed to use, edit and modify any map created with mapchart.net for publication freely by adding the reference to mapchart.net. The project of https://mapchart.net/ is licensed under a Creative Commons Attribution-ShareAlike 4.0 International License

*P. cynomolgi* mono-infection cases were predominantly in humans and *Anopheles* vectors, while an almost equal number of cases were reported in macaques. The proportion of *P. cynomolgi* cases in humans, *Anopheles*, and macaques per Southeast Asian country is shown in Fig. 2. The results showed that *P. cynomolgi* infection in humans was demonstrated in Malaysia and Cambodia. *P. cynomolgi* infection in *Anopheles* was demonstrated in Vietnam and Malaysia. *P. cynomolgi* infection in macaques was demonstrated in Malaysia, the Philippines, Indonesia, Cambodia, Laos, and Singapore. Human infection with *P. cynomolgi* was reported in Cambodia (*n* = 13) and Malaysia (*n* = 8). *Anopheles* infections with *P. cynomolgi* were reported in Vietnam (*n* = 20) and Malaysia (*n* = 8), while the highest numbers of macaques infected with *P. cynomolgi* were reported in Malaysia (*n* = 125) and Singapore (*n* = 66). The majority of the reports of infected macaques in Singapore (*n* = 40) did not identify whether the cases were mono or mixed infections. Studies on the presence of both vectors and natural hosts also reported human infection cases, as was the case in Malaysia (Fig. 3). *P. cynomolgi* infections in humans were reported in Cambodia and Malaysian Borneo, while *P. cynomolgi* infections in macaques were reported in the Philippines, Malaysia, Laos, Singapore, and Indonesia. *P. cynomolgi* infections in *Anopheles* mosquitoes were reported in southern Vietnam and Malaysian Borneo.

### The proportion of *P. cynomolgi* infection in humans

**Fig. 4:**
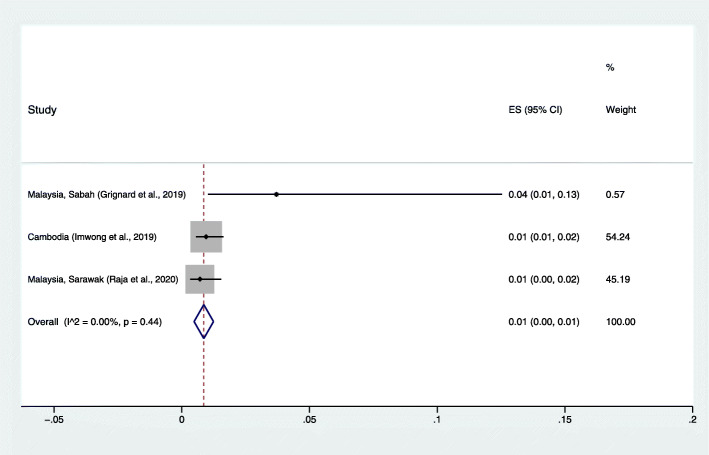
The estimated proportion of *P. cynomolgi* malaria in humans. ES: estimated proportion, overall: overall proportion, I^2^: level of heterogenicity, p: *p*-value less than 0.05 is statistically significant

The pooled prevalence of *P. cynomolgi* in humans was very low (< 0.1%). Because a low prevalence of *P. cynomolgi* in humans was reported in the included studies, the pooled proportion of *P. cynomolgi* compared to *Plasmodium* species infections in humans was estimated. The results demonstrated that the pooled proportion of naturally acquired *P. cynomolgi* was 1%, with low heterogeneity (95% CI: 0.1%, I^2^: 0%). The highest proportion of *P. cynomolgi* infection in humans was demonstrated in Northern Sabah (4, 95% CI: 1–13%) [18] (Fig. 4).

### The proportion of *P. cynomolgi* parasitemia in mosquitoes

**Fig. 5:**
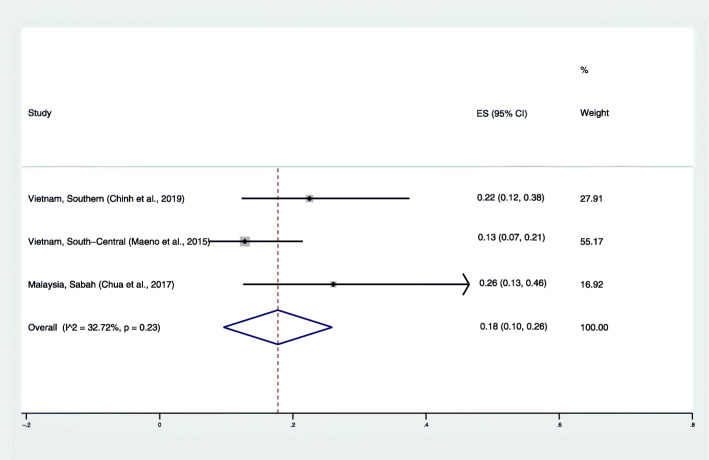
The estimated proportion of *P. cynomolgi* in vectors. ES: estimated proportion, overall: overall proportion, I^2^: level of heterogenicity, p: *p*-value less than 0.05 is statistically significant

The pooled prevalence of *P. cynomolgi* in mosquitoes was very low at 0–1%. Because a low prevalence of *P. cynomolgi* in mosquitoes was reported in the included studies, the pooled proportion of *P. cynomolgi* compared to *Plasmodium* species infections in mosquitoes was estimated. The results demonstrated that the pooled proportion of *P. cynomolgi* infecting mosquitoes was 18%, with low heterogeneity (95% CI: 10–26%, I^2^: 32.7%) (Fig. 5). The highest proportion of *P. cynomolgi* infection in mosquitoes was demonstrated in northern Sabah (26, 95% CI: 13–46%) [22].

### The prevalence and proportion of *P. cynomolgi* infection in macaques

**Fig. 6:**
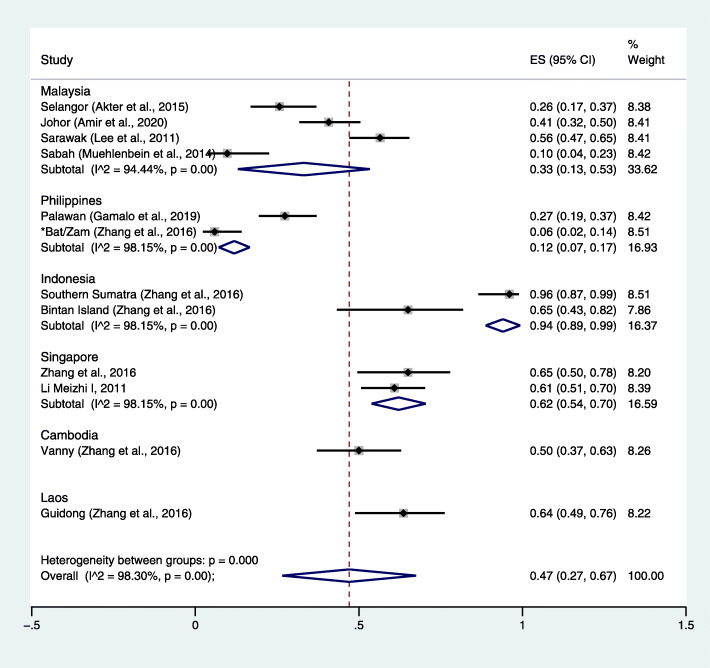
The estimated prevalence of *P. cynomolgi* malaria in macaques. ES: estimated prevalence, overall: overall prevalence, I^2^: level of heterogenicity, p: *p*-value less than 0.05 is statistically significant

**Fig. 7:**
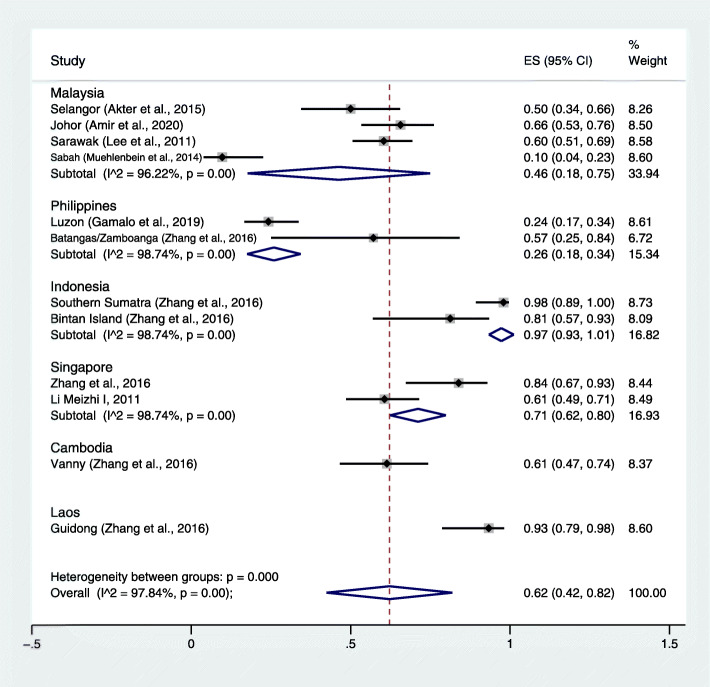
The estimated proportion of *P. cynomolgi* malaria in macaques. ES: estimated proportion, overall: overall proportion, I^2^: level of heterogenicity, p: *p*-value less than 0.05 is statistically significant

The pooled prevalence of *P. cynomolgi* compared to all macaques investigated was estimated. The results demonstrated that the pooled prevalence of *P. cynomolgi* infection in macaques was 47%, with high heterogeneity (95% CI: 27–67%, I^2^: 98.3%) (Fig. 6). The highest prevalence of *P. cynomolgi* infection in macaques was demonstrated in Indonesia (94, 95% CI: 89–99%) [17] and Singapore (62, 95% CI: 54–70%). The pooled proportion of *P. cynomolgi* compared to all malaria-positive samples was also estimated. The results demonstrated that the pooled proportion of *P. cynomolgi* infecting macaques was 67%, with high heterogeneity (95% CI: 42–82%, I^2^: 97.84%) (Fig. 7). The highest proportion of *P. cynomolgi* infection in macaques was demonstrated in Indonesia [17], while the lowest proportion of *P. cynomolgi* infection in macaques was demonstrated in the Philippines [13, 17].

### Streamlined publication years

**Fig. 8:**
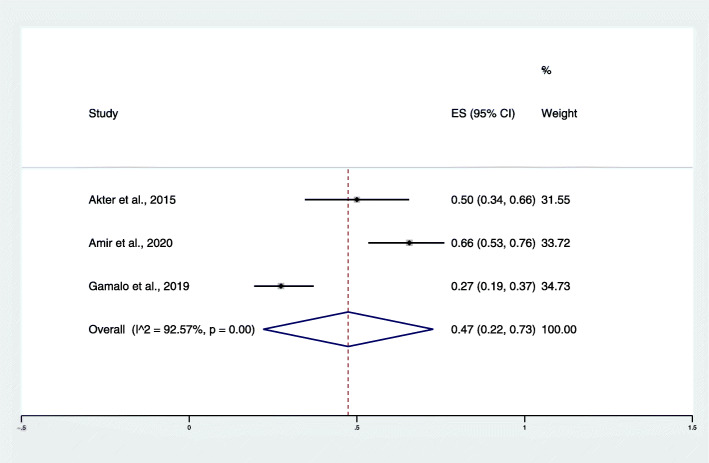
The pooled proportion of *P. cynomolgi* infection in macaques during 2015–2020. ES: estimated proportion, overall: overall proportion, I^2^: level of heterogenicity, p: *p*-value less than 0.05 is statistically significant

To reduce a large variance in the pooled proportion of *P. cynomolgi* infection between the three hosts and to describe the actual prevalence of *P. cynomolgi* infection in the past and present day, the cut-off point for publication years was streamlined for the purpose of this study. The year of studies was divided into studies reporting *P. cynomolgi* infection before 2013 and after 2013. Among the studies conducted before 2013, no studies reported the prevalence of *P. cynomolgi* infection in humans, while the study of Maeno et al. [23] during 2010–2013 demonstrated a pooled proportion of *P. cynomolgi* infection in mosquitoes of 13% (95% CI: 7–21%). The pooled proportion of *P. cynomolgi* infection in macaques was demonstrated by Lee et al. [15] during 2004–2008 at 56% (95% CI: 47–65%). Among studies conducted in and after 2013, the pooled proportion of *P. cynomolgi* infection in humans was estimated from three studies [18–20] during 2013–2017 at 1% (95% CI: 0.1%, I^2^: 0%). The pooled proportion of *P. cynomolgi* infection in mosquitoes was demonstrated by Chua et al. [22] during 2013–2014 at 26% (95% CI: 13–46%). In addition, the pooled proportion of *P. cynomolgi* infection in macaques during 2015–2020 was demonstrated at 47% (95% CI: 22–73%, I^2^: 92.57%) (Fig. 8).

## Discussion

The successful transmission of zoonotic malaria is highly dependent on the bionomics and distribution of competent vectors and natural hosts. Humans and macaques live in and share overlapping spaces, particularly those who work in farming or agriculture near forests or tourists who come to visit areas where macaques are endemic. These close contacts and sharing of geographic distributions between humans and macaques and the presence of suitable vectors can lead to malarial disease transmission between host species. The increase in zoonotic malaria before the discovery of *P. cynomolgi* was primarily found in the occurrence of *P. knowlesi* malaria. *P. knowlesi* infection in humans is driven by multiple factors, including anthropogenic land-use factors leading to changes in the transmission pattern of the parasite between macaque reservoirs, vectors, and humans, as observed in Malaysian Borneo [24]. Deforestation for palm oil plantations or other clearing activities resulted in the loss of natural habitats for macaques, which led to their close contact with human settlements and increased incidence of *P. knowlesi* infection in humans [25].

The present study demonstrated a high prevalence and density of *P. cynomolgi* in macaques (47%), particularly in Indonesia [17] and Singapore [14, 17]. Interestingly, half of the *P. cynomolgi* infections in macaques were mixed infections with other *Plasmodium* species, and among the mixed infections, triple and quadruple infections were the most common types of mixed infections observed. It should be noted that mono-infection cases were predominantly in humans and *Anopheles* vectors, while an almost equal number of cases were reported in macaques. More interestingly, *P. cynomolgi* was likely to be a mixed infection with *P. inui* (90.4%), *P. coatneyi* (51.4%), and *P. knowlesi* (45.9%). The latter seems to support the similarity of transmission between *P. cynomolgi* and the more well-known zoonotic malaria caused by *P. knowlesi*. Zoonotic malaria caused by *P. knowlesi* infections was described and focused on in Sarawak, Malaysian Borneo, in 2004 [1], and later, it became a more common cause and was recognized as the fifth human malaria parasite throughout Southeast Asia [2]. Similar to *P. knowlesi* infection, naturally acquired *P. cynomolgi* infection in Malaysia was reported 10 years later in 2014 in endemic cases of people who lived in the same area with long-tailed macaques [6]. From that time to 2020, a tourist who traveled to Surat Thani Province, Thailand, was naturally infected with *P. cynomolgi* [26]. Then, naturally acquired *P. cynomolgi* infections in humans were detected in other parts of Southeast Asia, including Malaysia and Cambodia [18–20]. Moreover, when comparing the pooled proportion of *P. cynomolgi* infections in three different hosts in the studies conducted before 2013 and after 2013, the pooled proportion of *P. cynomolgi* infections in mosquitoes was higher in studies conducted after 2013. Nevertheless, the pooled proportion of *P. cynomolgi* infections in macaques was lower in studies conducted after 2013. The results of these streamlined publication years suggested that *P. cynomolgi* infections in humans might be due to the proximity of suitable vectors and monkeys, leading to conditions favorable for interspecies transmission, as demonstrated in cases of *P. knowlesi* infection [11].

Figure 3 shows that regions with the presence of both vectors and natural hosts also reported human infection cases, as was the case in Malaysia. However, human transmission in Cambodia seemed to have been caused by the presence of macaques in the country and its proximity to southern Vietnam, which reported the most cases of infected *Anopheles* vectors. Although cases in macaques have been reported in the Philippines, Laos, Singapore, and Indonesia, there is a curiosity as to why there is a lack of reports on infected *Anopheles* mosquitoes and humans. The results of the present study indicated that in areas where a high prevalence of *P. cynomolgi* was seen in macaques, sustained public information and advocacy in the affected areas is still necessary even if public advocacies have already been performed since 2014 [27–30].

Although a high prevalence of *P. cynomolgi* in macaques was demonstrated by the included studies, the prevalence of *P. cynomolgi* in mosquitoes was very low at 0–1%, and the proportion of *P. cynomolgi* compared to all *Plasmodium* species detected in mosquitoes was 18%. Therefore, the transmission of *P. cynomolgi* from macaques to mosquitoes should be limited by the bite rates, the susceptibility of *Anopheles* vectors, and the parasite density of the primate host. The contrast between a high prevalence of *P. cynomolgi* in macaques and a lower parasitemia of *Anopheles* mosquitoes should be due to the limited susceptibility of *Anopheles* vectors to harbor a lower parasitemia of *P. cynomolgi* in a specific environmental condition, which can lead to disease transmission, as changes in host preference, biting behavior, and adaptation of the mosquitoes to habitat changes could affect the transmission of zoonotic malaria in Southeast Asia. Although the proportion of *P. cynomolgi* infecting *Anopheles* mosquitoes was low, the importance of transmission through the infection of human hosts by the bite of the infected *Anopheles* mosquitoes should be monitored. In addition, although the pooled proportion of naturally acquired *P. cynomolgi* in human hosts demonstrated by the included studies was 1%, the high density of infected mosquitoes, such as in Northern Sabah (26%) [22], could lead to a high prevalence of naturally acquired *P. cynomolgi* in humans, as demonstrated in Northern Sabah (4%) [18].

*P. cynomolgi* infection in humans was observed to be asymptomatic and submicroscopic [18, 19]. This implied that the mono-infection of *P. cynomolgi* was very low in parasite density despite the mixed infection with *P. vivax* as demonstrated by Imwong et al. [19]. The morphological life cycle of relapse and the genetic similarities of *P. cynomolgi* and *P. vivax* could lead to undetected or undiagnosed *P. cynomolgi* cases in Southeast Asia. Most of the cases with suspected malaria admitted to the hospital were identified by the microscopic method, which has a low sensitivity and specificity to differentiate *P. cynomolgi* from *P. vivax*. In addition, the misidentification or missed detection of submicroscopic parasitemia by *P. cynomolgi* might lead to under-reported cases. Moreover, failure to treat *P. cynomolgi* malaria can lead to malarial recurrences, as observed in two individuals from Charkrya and Ou Treng, Cambodia [19]. Even in symptomatic infections of *P. cynomolgi,* such as in the mixed infection of *P. cynomolgi* and *P. knowlesi,* as reported in the study by Raja et al. [20], the parasite density ranged from 213 to 84,299 parasites per milliliter. Moreover, the most recent study with enrolled febrile patients at malaria clinics or local hospitals in Thailand demonstrated that most of the patients with *P. cynomolgi* mixed infection with *P. vivax*, *P. falciparum* or both *P. vivax* and *P. knowlesi* had parasitemia less than 10,000 parasites/μL (< 0.2% parasitemia) as demonstrated by Putaporntip et al. [31]. The low proportion of *P. cynomolgi* detected in humans by three studies [18–20] might be due to the use of less-sensitive molecular protocols, as they used nested PCR amplifying the *SSU rRNA* gene for the detection of malaria parasites. PCR amplification of the malarial *cytochrome oxidase* gene is superior to PCR amplification of *SSU rRNA,* as *cytochrome oxidase* is a mitochondrial genome that is found at approximately 20–150 copies per parasite, whereas *SSU rRNA* is found at approximately 4–8 copies per parasite [32, 33].

The natural infection of *P. cynomolgi* in humans is similar to the infection of the natural host of long-tailed or pig-tailed macaques since mixed infections of more than one species in a single host were observed in both humans and macaques. Nevertheless, the dominance of *P. knowlesi* over *P. cynomolgi,* as demonstrated by Raja et al. [20], might be due to the differences in the developmental cycles of the two species. *P. cynomolgi* has both an exoerythrocytic cycle in the liver and erythrocytic cycle in red blood cells. This means that the incubation period for *P. knowlesi* is shorter by approximately 9–12 days than that of *P. cynomolgi,* which is between 15 and 37 days [7]. Furthermore, the erythrocytic cycle of *P. cynomolgi* (48 h) is longer than that of *P. knowlesi* (24 h) [34]. Therefore, *P. cynomolgi* mixed infection with *P. knowlesi* might occur simultaneously in a single mosquito bite harboring two parasite species. The simultaneous infection of *P. cynomolgi* with *P. knowlesi* in a single mosquito was demonstrated in a study by Chua et al. [22].

The present study had limitations. First, the number of studies identifying *P. cynomolgi* in humans and mosquitoes was limited, resulting in an imprecise estimate of the pooled prevalence or pooled proportion and of the clinical characteristics of human hosts. Second, the Northern Sabah study [18] demonstrated a lower NOS scale (2 stars) as poor comparability against the other studies which used molecular detection methods but retained in the present analysis. Therefore, the pooled proportion of *P. cynomolgi* infection in humans requires careful interpretation. Further studies and continued surveillance of *P. cynomolgi* throughout Southeast Asia and neighbouring regions may be necessary through the use of sensitive molecular protocols to obtain accurate and relevant data for the detection and reporting of this emerging zoonotic malaria. Moreover, it is essential to further explore the biology of *P. cynomolgi,* the possibility of relapses/recurrences, and asymptomatic infections, which have a direct impact on the epidemiology of malaria.

## Conclusion

In conclusion, *P. cynomolgi* could continue to be a public health concern in Southeast Asian countries, as the natural habitat of the natural hosts and vectors and the evolution of the *P. cynomolgi* parasite itself could drive the transmission of this neglected but emerging disease. In addition, the environmental and climatic changes experienced worldwide could affect the transmission dynamics of *P. cynomolgi* as an emerging cause of malaria in humans. Further studies using molecular and multidisciplinary approaches to search for *P. cynomolgi* infection in humans, vectors, and natural hosts are necessary if human infections with *P. cynomolgi* do become public health concerns.

## Supplementary Information

**Additional file 1: Table S1.** Quality of the included studies

## Data Availability

All data related to the present study are available in this manuscript.
