# Supplementary material for: Preliminary review on the prevalence, proportion, geographical distribution, and characteristics of naturally acquired Plasmodium cynomolgi infection in mosquitoes, macaques, and humans: a systematic review and meta-analysis
Source: BMC Infect Dis. 2021 Mar 12;21:259. doi: 10.1186/s12879-021-05941-y (PMC7953546; doi:10.1186/s12879-021-05941-y)
Supplement: Supplementary file 1 — Additional file 1: Table S1. Quality of the included studies [file 12879_2021_5941_MOESM1_ESM.docx]

**Preliminary review on the prevalence, proportion, geographical distribution, and characteristics of naturally acquired *Plasmodium cynomolgi* infection in mosquitoes, macaques, and humans: a systematic review and meta-analysis**

Manas Kotepui^1*^, Frederick Ramirez Masangkay^2^, Kwuntida Uthaisar Kotepui^1^, Giovanni De Jesus Milanez^2^

^1^Medical Technology, School of Allied Health Sciences, Walailak University, Thasala, Nakhon Si Thammarat, Thailand

^2^Department of Medical Technology, Institute of Arts and Sciences, Far Eastern University-Manila, Manila, Philippines

Authors’ Email Addresses:

**^*^Corresponding Author**: Manas Kotepui; manas.ko@wu.ac.th, +66954392469

Frederick Ramirez Masangkay; frederick_masangkay2002@yahoo.com

Kwuntida Uthaisar Kotepui; kwuntida.ut@wu.ac.th

Giovanni De Jesus Milanez; gmilanez@feu.edu.ph

**Table S1.** Quality of the included studies

| **No.** | **Author, year, reference number** | **Selection** | | | | **Compatibility** | **Exposure** | | | **Total score (3)** | **Rating (High, moderate, low quality)** |
| --- | --- | --- | --- | --- | --- | --- | --- | --- | --- | --- | --- |
|  |  | **Is the Case Definition Adequate?** | **Representativeness of the Cases** | **Selection of Controls** | **Definition of Controls** |  | **Ascertainment of Exposure** | **Same method of ascertainment for cases and controls** | **Non-Response Rate** |  |  |
| 1. | Grignard et al., 2019 | 🟑 |  | NA | NA | NA | 🟑 | NA | NA | 2 | High |
| 2 | Imwong et al., 2019 | 🟑 | 🟑 | NA | NA | NA | 🟑 | NA | NA | 3 | High |
| 3. | Raja et al., 2020 | 🟑 | 🟑 | NA | NA | NA | 🟑 | NA | NA | 3 | High |
| 4. | Akter et al., 2015 | 🟑 | 🟑 | NA | NA | NA | 🟑 | NA | NA | 3 | High |
| 5. | Amir et al., 2020 | 🟑 | 🟑 | NA | NA | NA | 🟑 | NA | NA | 3 | High |
| 6. | Lee et al., 2011 | 🟑 | 🟑 | NA | NA | NA | 🟑 | NA | NA | 3 | High |
| 7. | Muehlenbein et al., 2014 | 🟑 | 🟑 | NA | NA | NA | 🟑 | NA | NA | 3 | High |
| 8. | Gamalo et al., 2019 | 🟑 | 🟑 | NA | NA | NA | 🟑 | NA | NA | 3 | High |
| 9. | Zhang et al., 2016 | 🟑 | 🟑 | NA | NA | NA | 🟑 | NA | NA | 3 | High |
| 10. | Li Meizhi I, 2011 | 🟑 | 🟑 | NA | NA | NA | 🟑 | NA | NA | 3 | High |
| 11. | Chinh et al., 2019 | 🟑 | 🟑 | NA | NA | NA | 🟑 | NA | NA | 3 | High |
| 12. | Maeno et al., 2015 | 🟑 | 🟑 | NA | NA | NA | 🟑 | NA | NA | 3 | High |
| 13. | Chua et al., 2017 | 🟑 | 🟑 | NA | NA | NA | 🟑 | NA | NA | 3 | High |

🟑 A star rating
